# Yeasts Acquire Resistance Secondary to Antifungal Drug Treatment by Adaptive Mutagenesis

**DOI:** 10.1371/journal.pone.0042279

**Published:** 2012-07-31

**Authors:** David Quinto-Alemany, Ana Canerina-Amaro, Luís G. Hernández-Abad, Félix Machín, Floyd E. Romesberg, Cristina Gil-Lamaignere

**Affiliations:** 1 Unidad de Investigación. Hospital Universitario Nuestra Señora de Candelaria, Santa Cruz de Tenerife, Spain; 2 Chemistry Department, The Scripps Research Institute, La Jolla, California, United States of America; Louisiana State University, United States of America

## Abstract

Acquisition of resistance secondary to treatment both by microorganisms and by tumor cells is a major public health concern. Several species of bacteria acquire resistance to various antibiotics through stress-induced responses that have an adaptive mutagenesis effect. So far, adaptive mutagenesis in yeast has only been described when the stress is nutrient deprivation. Here, we hypothesized that adaptive mutagenesis in yeast (*Saccharomyces cerevisiae* and *Candida albicans* as model organisms) would also take place in response to antifungal agents (5-fluorocytosine or flucytosine, 5-FC, and caspofungin, CSP), giving rise to resistance secondary to treatment with these agents. We have developed a clinically relevant model where both yeasts acquire resistance when exposed to these agents. Stressful lifestyle associated mutation (SLAM) experiments show that the adaptive mutation frequencies are 20 (*S. cerevisiae* –5-FC), 600 (*C. albicans* –5-FC) or 1000 (*S. cerevisiae* – CSP) fold higher than the spontaneous mutation frequency, the experimental data for *C. albicans* –5-FC being in agreement with the clinical data of acquisition of resistance secondary to treatment. The spectrum of mutations in the *S. cerevisiae* –5-FC model differs between spontaneous and acquired, indicating that the molecular mechanisms that generate them are different. Remarkably, in the acquired mutations, an ectopic intrachromosomal recombination with an 87% homologous gene takes place with a high frequency. In conclusion, we present here a clinically relevant adaptive mutation model that fulfils the conditions reported previously.

## Introduction

Acquisition of resistance secondary to treatment both by microorganisms and by tumor cells is a major public health concern. This resistance can have a metabolic (eg. overexpression of efflux pumps) or genetic origin (through mutations). Several species of bacteria acquire resistance to various antibiotics (e.g. rifamycins, trimethoprim and β-lactams [1]–[4]) through stressful lifestyle-associated mutations (SLAM, [5]) using stress-induced responses (eg. SOS, RpoS, etc) that have an adaptive mutagenic effect. “Adaptive mutagenesis” has been defined as “a generic term for processes that allow **individual cells** of **nonproliferating** cell populations to acquire advantageous mutations and thereby to **overcome** the strong **selective pressure** of proliferation limiting environmental conditions” i.e. adapt to the environment [6].

Very little is known about the mechanisms that yeasts use to adapt to environmental stress. So far, adaptive mutagenesis in yeast has only been described when the stress is nutrient deprivation (for a review, see [6]). The aim of this work was to develop a broader adaptive mutation model with clinical relevance. We used *Saccharomyces cerevisiae* and *Candida albicans* as model organisms, and as environmental stress the antifungal agents 5-fluorocytosine (flucytosine, 5-FC) and caspofungin (CSP). We selected 5-FC because of the very high occurrence of secondary resistance in patients that creates the need for its administration in combination with another drug (typically amphotericin B or fluconazole) [7], [8], and CSP because of its relevance as one of the newest antifungal agents.

In *S. cerevisiae*, cellular uptake of 5-FC relies on a purine-cytosine permease encoded by FCY2. Once inside the cell, 5-FC is converted to 5-fluorouracil (5-FU) through the action of a cytosine deaminase, encoded by FCY1. The uracil phosphoribosyltransferase (UPRTase) encoded by FUR1 then converts 5-FU into 5-fluorouridine-5′-monophosphate (5-FUMP), which is then further phosphorylated by kinases to yield the uracil di- and triphosphate analogs, 5-fluorouridine diphosphate (5-FUDP) and triphosphate (5-FUTP). 5-FUTP is incorporated into fungal RNA in place of uridylic acid, altering the aminoacylation of tRNA, disturbing the aminoacid pool and inhibiting protein synthesis [8], [9]. Alternatively, 5-FUDP may be converted to 5-fluorodeoxyuridine diphosphate (5-FdUDP) through the action of RNR. 5-FdUMP inhibits DNA synthesis through the inhibition of thymidylate synthase (encoded by CDC21). Resistance to 5-FC has been proposed to occur through mutations in the afore-mentioned genes, FCY2, FCY1, FUR1, CDC21, or even through mutations that lead to the up-regulation of the pyrimidine synthesis [8], [10]. Nevertheless, since 5-FC interferes with DNA and RNA synthesis [8] and one might argue that it has a mutagenic effect per se (albeit not described in the literature), we have validated our results with a drug that has a completely different mode of action, CSP. This is an echinocandin that inhibits cell-wall synthesis (by non-competitive inhibition of the β(1–3) glucane sythase) and does not penetrate the cell membrane, and thus would not directly interfere with nucleotide metabolism [10]. Mutations in the coding subunits of the β(1–3) glucane sythase, FKS1 and FKS2, have been shown to cause resistance in patient-derived strains. In this work we describe how the acquisition of resistance secondary to treatment with either drug takes place in vitro and we show how this acquisition fulfills the previously described requirements for an adaptive mutagenesis model in yeasts [6].

## Materials and Methods

### Yeast Strains and Media

Strains used in this study are *S. cerevisiae* BY4741 and *C. albicans* SC5314. *S. cerevisiae* and *C. albicans* were grown at 30 or 37°C respectively, in YPD medium (1% yeast extract, 2% peptone, 2% dextrose) or synthetic complete medium (SC; 0.67% yeast nitrogen base, 2% glucose) supplemented with aminoacids as described in Burke, et al. [11]. Solid media contained 2% agar. 5-FC-resistant mutants (5-FC^r^) were selected on SC containing 100 µg/ml 5-FC. CSP-resistant mutants (CSP^r^) were selected on SC containing 0.72 µg/ml CSP.

**Table 1:** Primers used for amplification and sequencing of the genes causing 5-FC resistance.

Name	Oligo sequence	Annealing (°C)	
**Gene amplification primers and conditions**			
Fcy2_f	ATGGAACGGCCTCAAGGAACT		57
Fcy2_r	TGATACATGACGTGAAATGTG C		57
FCY1_af	GTTTTCTATTGCCATTTTTATCG		55
FCY1_ar	ACCTGAACACCGACGAAGAC		55
Fur1_af	GAGGAACCGATTGGCAGAGC		59
Fur1_ar	TCAAGATGGTGTTCGGGTGTG		59
CDC21_af	GCTTCTTTCCCCTCTCGTC		57
CDC21_ar	TCTTTTTGCCCTGGTGTTCC		57
**Gene sequencing primers and conditions**			
Fcy2_F0	GCATATAAAACATCCTATCC		50
Fcy2_F1	TTTGGGTGCCTTAGGAC		50
Fcy2_F2	AAGGTGGTGAATGGGTAG		50
Fcy2_R1	CCCAACCGACACAAGC		50
Fcy2_R2	TAGGAACCAGGATAGCAT		50
Fcy2_R3	GAAATGTGCACGGGGAAATGA		50
FCY1_ars	TTTCAAGTCTTCCCTAGTAGTG		50
FCY1_sf	TAGTGACCTATGGTGTG		50
Fur1_sf	AAGCTGCCTCAAAAGAG		50
CDC21_sf	AGTATCAAGGAGAGAGC		50
CDC21_sr	TTTCTCCTCGTGCTGTC		50

### Reagents

Caspofungin acetate was purchased from Merck Sharp & Dohme Ltd., UK, 5-Fluorocytosine from Alfa Aesar GmbH, Germany, Taq DNA polymerase kit from VWR, Denmark, low melting agarose for CHEF from Promega Corp, Madison, WI and agarose D1 low EEO from Pronadisa, Spain. Sequencing was performed using Big Dye 3.1 (Applied Biosystems, Foster City).

### Minimum Inhibitory Concentration

The minimum inhibitory concentration (MIC) of drugs in solid SC media was determined by pouring 1 ml SC medium containing several dilutions of either 5-FC or CSP on each well of a 24-well plate. For 5-FC, 1.7·10^4^ cells were inoculated on each well (to have the same cell density on the agar surface as 5·10^5^ cells on 90 mm petri dishes, used in SLAM experiments described below). For CSP, 1.7·10^5^ cells were inoculated on each well (same cell density as 5·10^6^ cells on 90 mm petri dishes). After 48 and 72 hours, growth on the surface of the agar was evaluated. The MIC was determined as the smallest drug concentration where the cells formed less than 3 colonies on the surface (spontaneous resistant cells).

**Figure 1:**
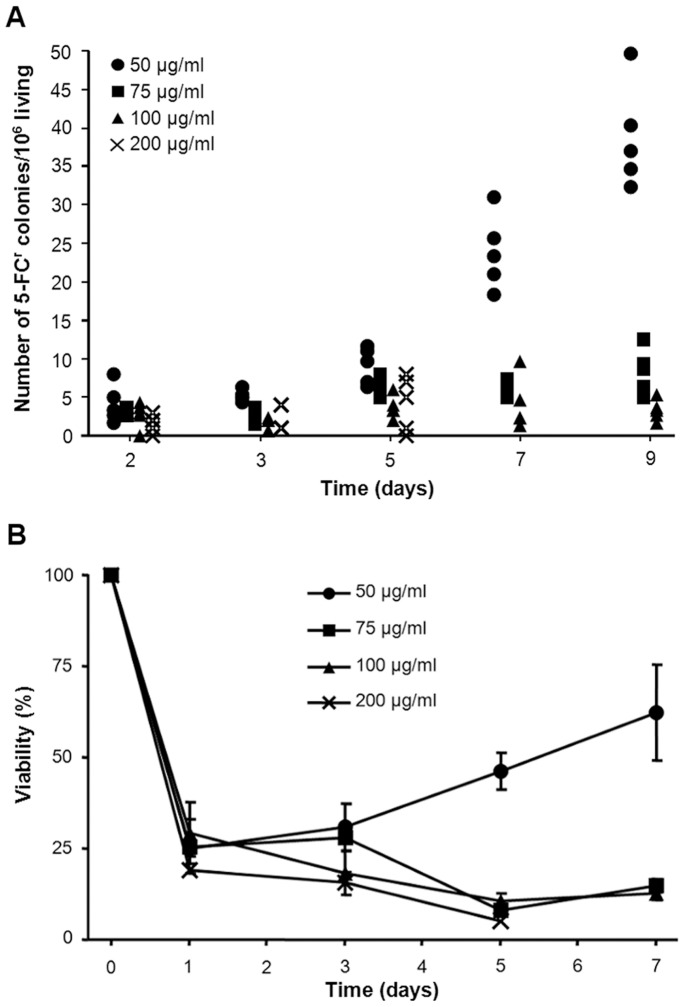
Effect of various concentrations of 5-FC in *S. cerevisiae*. Behaviour of *S. cerevisiae* during prolonged incubation on media containing 50 µg/ml (circles), 75 µg/ml (squares), 100 µg/ml (triangles) or 200 µg/ml (cross) 5-FC. (**A**) Kinetics of the appearance of *S. cerevisiae* resistant to 5-FC. Cells (5·10^5^) were used to inoculate 5-FC supplemented SC agar medium. Plates were incubated in a moisture chamber at 30°C and scored bidaily for the appearance of 5-FC^r^ colonies. The frequency of 5-FC^r^ cells was calculated as the number of drug resistant colonies observed each day for a given clone divided by the number of cells present on the plates two days earlier (moment when one cell mutated and began giving rise to the visible colony counted). (**B**) 5-FC leads to some cell death in a concentration dependent manner. Viable colony forming units were determined by recovering cells from 5-FC-containing plates and replating on permissive medium. Survival is shown relative to the number of viable cells present 3 hours after plating on 5-FC (as explained in the Methods section) and is the mean and standard error for 5 independent subpopulations.

### Determination of Adaptive Mutation Frequencies: SLAM Experiments

Stressful-lifestyle-associated mutation (SLAM) experiments were performed as follows: Individual colonies (originally descended from a single cell) were suspended in YPD and incubated at 30°C (*S. cerevisiae*) or 37°C *(C. albicans)* to obtain different subpopulations. When these cultures reached a density of approximately 10^8^ cells/ml the cell concentration was adjusted to 5·10^6^ (5-FC) or 5·10^7^ (CSP) cells/ml with 0.9% sterile NaCl solution. Each subpopulation was then plated on 2 solid SC medium plates with antifungal drug at a density of 5·10^5^ (5-FC) or 5·10^6^ (CSP) cells per 90-mm-diameter dish. A moisturized chamber at either temperature was used to avoid desiccation of the plates. Only those cells already harboring mutations that confer resistance at the time of plating were able to continue proliferation and form colonies on the drug containing medium right after plating. Based on the growth of the 5-FC^r^ strains *fcy1*Δ and *fcy2*Δ, visible colonies of resistant mutants are visible within 24–60 h. Following the appearance of colonies formed by the pre-existing mutants (*i.e.* after the first 3 days of selection for drug resistance), additional resistant mutants continued to appear during prolonged incubation of the plates. These newly arising colonies were scored bidaily for 9 days post plating. The number of viable cells during the course of the experiment was determined by excising identical pieces of surface (plugs) on drug-containing SC plates. Cells from the agar plugs were plated at an appropriate dilution on YPD plates, and colony forming units were counted after 5 days to ensure that any living cell present would form a visible colony. To calculate spontaneous mutation frequencies (referred to as “day 3”), colonies appeared on or before day 3 were divided by the number of viable cells counted 3 hours post-exposure, providing the cells ample time to finish whatever cycle they had started during YPD incubation and to incorporate 5-FC into the cell and start its action. To calculate adaptive mutation frequencies the number of mutant colonies was normalized by the number of viable cells present on the plate when the mutation took place (two days before). Therefore, the number of mutant colonies visible on days 5, 7 or 9 was divided by the number of viable cells on days 3, 5 or 7. Frequencies presented are cumulative starting on day 5 as customary in the pertinent literature.

**Figure 2:**
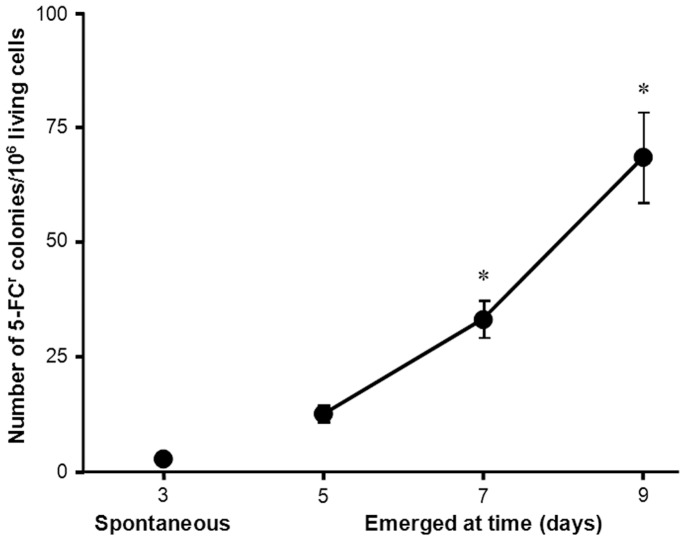
Kinetics of acquisition of resistance by *S. cerevisiae* to 5-FC. Kinetics of the acquisition of resistance by *S. cerevisiae* during prolonged incubation on medium containing 100 µg/ml 5-FC. Cells (5·10^5^) were used to inoculate 5-FC supplemented SC agar medium and SLAM experiments performed as described in the Methods section. Mean and standard error for 29 independent subpopulations. * indicates p<0.001 as evaluated by ANOVA with Bonferroni post-hoc test.

According to the Luria-Delbruck model [12], if the cells acquire a spontaneous mutation when growing in the liquid medium before being plated on stressful conditions, a mutation acquired early in the liquid culture will possibly take place, leading to an abnormal amount of colonies (Jackpot). Subpopulations that displayed a jackpot on day 3 were removed from the experiments because they provide a fortuitous deviation of the mean and due to the methodological problem posed, since an excessive number of resistant colonies on the agar surface prevent us from extracting resistant-cell free plugs to calculate the number of viable cells.

### Reconstruction Experiments

To verify the mutational origin of the late-arising colonies as opposed to a metabolic origin, 192 5-FC^r^ *S. cerevisiae* and 96 5-FC^r^ *C. albicans* colonies arising at days 5 or later were subcultured on SC+5-FC plates, then in liquid YPD and then again on SC+5-FC plates. In 98% of the cases, the colonies grew within 48–60 hours. Ninety six CSP^r^ *S. cerevisiae* colonies arising at day 5 or later were subcultured on SC+CSP plates, then in liquid YPD and then again on SC+CSP plates. In 97% of the cases, the colonies grew within 48–60 hours.

**Figure 3:**
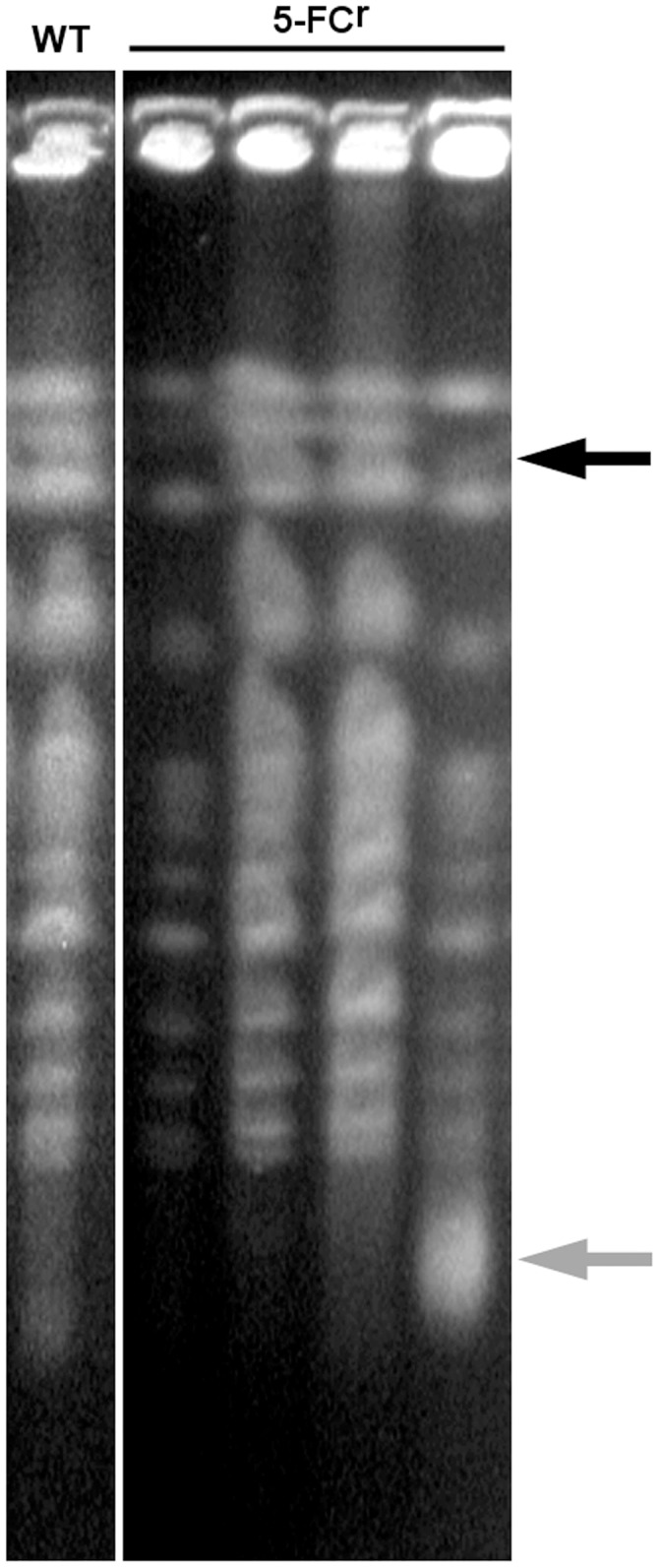
Analysis of gross chromosomal rearrangements in *S. cerevisiae* resistant to 5-FC by pulse-field gel electrophoresis. CHEF of the chromosomes of a wild-type (WT) and 4 of the secondary 5-FC^r^ strains. The black arrow indicates the only rearrangement found, an apparent deletion in chromosome IV concomitant with a new diffuse band of low molecular weight (grey arrow). Run conditions: 1% agarose gel in 0.5× TBE buffer and run at 14°C for 24 h at 6 V/cm with an initial switching time of 60 seconds, a final of 120 seconds, and an angle of 120°.

### Sequence Analysis

Genomic DNA of randomly chosen late 5-FC^r^ colonies was prepared using glass beads, followed by phenol-chloroform-isoamyloalcohol and ethanol precipitation. Fragments encompassing the complete genes were amplified and sequenced in-house using an ABI 3500 Genetic Analyzer (applied Biosystems, Foster City). Table 1 shows a list of primers and conditions used.

### Cell Cycle Analysis by FACS

Flow cytometry analysis was carried out as described before [13]. Briefly, for each time point, resistant colonies were excised from one 90 mm diameter plate and the remaining non-resistant cells were washed off with 70% EtOH and stored at 4°C for 1 to 15 days. After RNase and proteinase K treatment, they were stained with 50 µg/ml propidium iodide (Sigma-Aldrich Chemie Gmbh, Germany) in a BD FACScalibur flow cytometer, adjusting the peaks for 1 N and 2 N with an asynchronous culture at 30°C before reading the samples.

### Microscopic Analysis

Time-lapse microscopy experiments were performed to evaluate the effect of 5-FC on cell morphology. For these experiments, one 5-FC^r^ (used as control for normal growth) and 2 WT subpopulations were plated on SC+5-FC medium as in SLAM assays, a 2×3 cm slice was cut out and placed on a glass slide. After microscopic examination of 7 fields (enough to count 300–500 cells at time 0), the slides were kept in a moisture chamber at 30°C for 12 days. Exactly the same fields were examined at specified times to observe cell division. To evaluate the percentage of cells that initiated cytokinesis (assessed as budding), 182 cells from 3 different fields at 3 hours post-exposure were followed through time. The percentage was calculated as the number of cells that initiated bud formation x100/182 within 7 days.

To examine nuclear division of the same subpopulations, for each time point, all the 5-FC^r^ colonies were excised from one 90 mm diameter plate and the remaining non-resistant cells were washed off with sterile H_2_O, collected and kept at −24°C. On the day of the experiment, cells were thawed, stained with 1 µg/ml DAPI (Sigma-Aldrich Chemie Gmbh, Germany) and examined under fluorescence. For optimal visualization and presentation of the data, the fluorescent field was colored in a gradient yellow – red – black from brightest toward darkest light intensities.

### CHEF by Pulse-field gel Electrophoresis (PFGE)

PFGE to see all yeast chromosomes was performed using a CHEF DR-III system (Bio-Rad) in a 1% agarose gel in 0.5× TBE buffer and run at 14°C for 24 h at 6 V/cm with an initial switching time of 60 seconds, a final of 120 seconds, and an angle of 120°. Ethidium bromide was used to visualize the chromosome bands in the gel.

**Table 2:** Effect of 5-FC on the spectrum of mutations in four genes that may be involved in drug resistance.

Day	Gene	Mutation				Type	Effect	Day	Gene	Mutation			Type	Effect	
2	*FCY1*	190 GC→AT	(V233→K)			TI	M	7	*FCY2*	410 CG→TA		(A136→V)	TI	M	
2	*FCY1*	107 GC→AT	(C205→STOP)			TI	N	7	*FCY2*	843 GC→AT	(W281→STOP)		TI	N	
2	*FCY1*	179 CG→AT	(T229→K)			TV	M	7	*FCY2*	1594 GC→CG		(G532→R)	TV	M	
2	*FCY1*	58 CG→AT	(E189→STOP)			TV	N	7	*FCY2*	410 CG→AT		(A137→E)	TV	M	
2	*FCY1*	58 CG→AT	(E189→STOP)			TV	N	7	*FCY2*	1311 CG→AT		(Y437→STOP)	TV	N	
2	*FCY1*	58 CG→AT	(E189→STOP)			TV	N	7	*FCY2*	308 CG→AT		(S102→STOP)	TV	N	
3	*FCY2*	FCY22 968–1470				R		7	*N.F.*						
3	*FCY2*	1226 TA→AT			(L657→STOP)	TV	N	9	*FCY2*	Δ657			D	F	
3	*FCY2*	1475 TA→AT			(Y493→STOP)	TV	N	9	*FCY2*	Δ155–157			D	F	
3	*FCY2*	1475 TA→AT			(Y493→STOP)	TV	N	9	*FCY2*	FCY22 1098–1143			R		
3	*FCY2*	1568 TA→GC			(L522→STOP)	TV	N	9	*FCY2*	FCY22 1098–1143			R		
5	*FCY1*	41 GC→CG			(G14→A)	TV	M	9	*FCY2*	FCY22 1098–1143			R		
5	*FCY2*	fcy2Δ				D		9	*FCY2*	FCY22 968–1143			R		
5	*FCY2*	FCY22 1053–1143				R		9	*FCY2*	453 GC→AT		(M151→I)	TI	M	
5	*FCY2*	FCY22 968–1143				R		9	*FCY2*	680 AT→GC		(Y227→C)	TI	M	
5	*FCY2*	FCY22 909–1143				R		9	*FCY2*	1136 AT→GC		(Y378→C)	TI	M	
5	*FCY2*	FCY22 1065–1143				R		9	*FCY2*	1205 TA→GC		(M401→R)	TV	M	
5	*FCY2*	FCY22 1014–1143				R		9	*FCY2*	1344 CG→AT		(Y448→STOP)	TV	N	
5	*FCY2*	FCY22 1089–1143				R		9	*N.F.*						
5	*FCY2*	FCY22 1103–1143				R		9	*N.F.*						
5	*FCY2*	448 CG→TA		(Q150→STOP)		TI	N			Double Mutants					
5	*FCY2*	1594 GC→CG			(G532→R)	TV	M	7	*FCY2*	FCY22 978–1143			R		
5	*FCY2*	842 GG→TA			(W280→L)	TV	M		*FCY1*	316 TA→CG		(C106→R)	TI	M	
5	*N.F.*							7	*FCY2*	FCY22 884–1074			R		
7	*FCY2*	Δ469–472				D	F		*FUR1*	518 AT→GC		(E173→G)	TI	M	
7	*FCY2*	fcy2Δ				D		7	*FCY2*	Δ1222–1602			D		
7	*FCY2*	1020 insertion A_2_				I	F		*FCY2*	1214 AT→CG		(N404→T)	TV	M	
7	*FCY2*	FCY22 1053–1143				R		7	*FCY2*	FCY22 913–1143			R		
7	*FCY2*	FCY22 1053–1143				R			*FCY2*	Δ909			D	F	
7	*FCY2*	FCY22 1065–1143				R		9	*FCY1*	258 GC→AT		(Syn)	TI	S	
7	*FCY2*	FCY22 263–565				R			*N.F.*						
7	*FCY2*	FCY22 378–693; 1443–1512				R		9	*FCY2*	FCY22 1053–1143			R		
7	*FCY2*	FCY22 844–1143				R			*FCY1*	437 TA→CG		(L146→P)	TV	M	
7	*FCY2*	FCY22 968–1143				R		9	*FCY2*	392 TA→GC		(Syn)	TV	S	
7	*FCY2*	FCY22 968–1143				R			*FCY1*	173 CG→AT		(S58→Y)	TV	M	
7	*FCY2*	FCY22 1089–1137				R		9	*FCY2*	FCY22 300–513			R		
7	*FCY2*	FCY22 1123–1137				R			*FCY2*	753 GC→AT		(Syn)	TI	S	
7	*FCY2*	FCY22 1104–1143				R		9	*FCY2*	945 insertion T_#_			I	F	
7	*FCY2*	FCY22 921–1143				R			*FCY2*	937 CG→AT		(L913→I)	TV	M	
7	*FCY2*	832 GC→AT			(A278→T)	TI	M	9	*FCY2*	453 GC→AT		(M151→I)	TI	M	
7	*FCY2*	914 TA→CG			(L305→F)	TI	M		*FCY2*	445 AT→CG		(Syn)	TV	S	

N.F.: No mutation found, Syn: Sinonimous mutation, D: deletion, I: Insertion, R: recombination, TI: transition, TV: transversion, M: missense, N: nonsense, S: silent, F: frameshift.

### Statistical Analysis

Mean and Standard Error of the Mean were calculated using the statistics package Instat V 3.10 by GraphPad Software, Inc. The significance of the differences between mutants emerged by day 3 (spontaneous) and those emerged later (P values, two-tailed 95% confidence value) were calculated using ANOVA with Bonferroni post-hoc test.

## Results

### Prolonged Exposure to 5-FC Increases the Mutation Frequency in *S. Cerevisiae*

We examined the behavior of 5 *S. cerevisiae* subpopulations during exposure to various concentrations of 5-FC through time (Fig. 1). *S. cerevisiae* colonies resistant to 5-FC (Fig. 1a) appeared after long-term incubation on drug-supplemented SC medium within a concentration window (e.g. too low, all cells survive; too high, invariably lethal, fig. 1b). The lowest concentration that inhibited growth (MIC) was 25 µg/ml, while the lowest concentration that was invariably lethal (MLC) was 400 µg/ml (16 fold the MIC). Thus, we selected 4 fold the MIC for the subsequent experiments.

**Figure 4:**
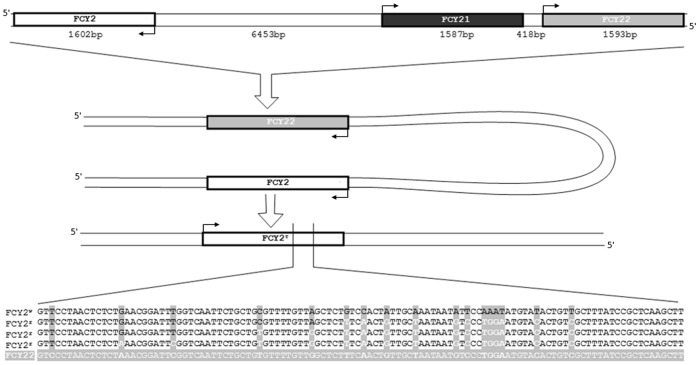
Intrachromosomal recombination of FCY2. Ectopic homologous recombination events found. FCY2, FCY21 and FCY22 are on chromosome V, at the specified distances. After folding, the recombination event takes place, yielding the FCY2^r^ sequences. The arrows mark the start codon in either the Watson or the Crick strands. FCY2^w^ is the wild-type FCY2 sequence; FCY2^r^ are the sequences of three chosen recombinant mutants, and FCY22 is the pseudogen FCY2 has recombined with.

Next, we evaluated the kinetics of the acquisition of resistance to 5-FC through time (Fig. 2). As expected, a few resistant colonies grew within 1 to 3 days (pre-existing or spontaneous), most likely due to spontaneous mutations that took place while growing on drug-free liquid medium. This occurred at a spontaneous mutation frequency of 3·10^−6^±3.9·10^−7^ mutants/cell. In these 6 SLAM experiments, comprising 29 subpopulations, we found a jackpot only once (see methods section).

Prolonged exposure of *S. cerevisiae* to fourfold 5-FC MIC resulted in the appearance of resistant colonies that continued to accumulate for longer than 16 days after initial exposure to the drug (long-term or acquired resistant). Experiments were terminated at day 9, when they reached a final frequency of 6.9·10^−5^±9.8·10^−6^ mutants/cell. These two frequencies (spontaneous vs. adaptive) were significantly different (p<0.001), reflecting the very different conditions in which mutations took place, and suggesting the possibility of different underlying mechanisms.

**Figure 5:**
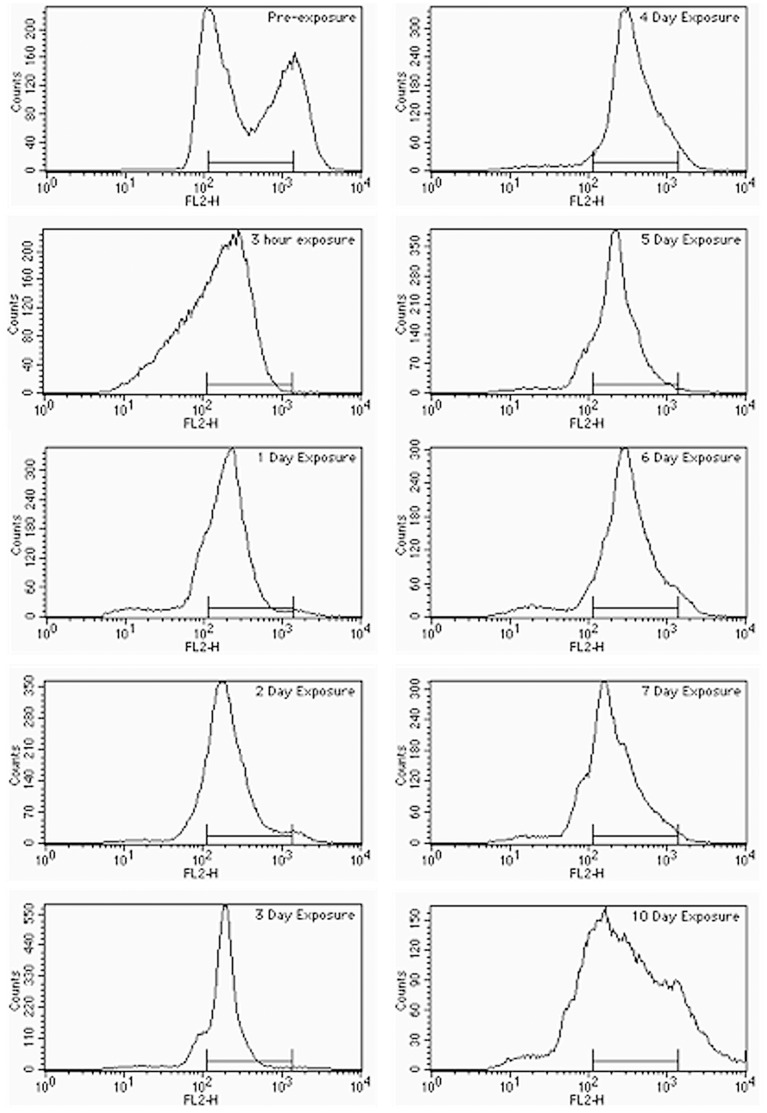
Effects of prolonged exposure to 5-FC on cell cycle progression. For each panel, resistant colonies were excised from one 150 mm diameter plate and the remaining non-resistant cells were washed off with 80% EtOH to measure DNA contents by FACS as described in Materials and Methods. In each panel the X axis represents the DNA content and the Y axis represents the number of cells. The FACS histograms measured at various times as specified in each panel.

Reconstruction experiments were also performed to examine the stability of acquired resistant phenotypes and to evaluate the possibility that those colonies came from slow-growing individual cells. Ninety eight percent of the 192 5-FC^r^ colonies examined grew within 48–60 hours (most of them were already visible before 48 hours). These experiments enabled us to rule out the hypotheses of slow-growing cells as origin of the late-arising colonies, and to establish the genetic (hereditary) source of the long-term (acquired) resistance.

### Analysis of the Spectrum of Mutations that Causes Resistance

To gain further insight into the hereditary source of the acquired resistance and the possible mechanisms for it, we examined whether the resistant clones had experienced gross chromosomal rearrangements and we analyzed the sequences of the four genes that seemed more likely to be involved: the permease *FCY2*, the deaminase *FCY1*, the ribosyl transferase *FUR1* and the TMP synthase *CDC21*.

**Figure 6:**
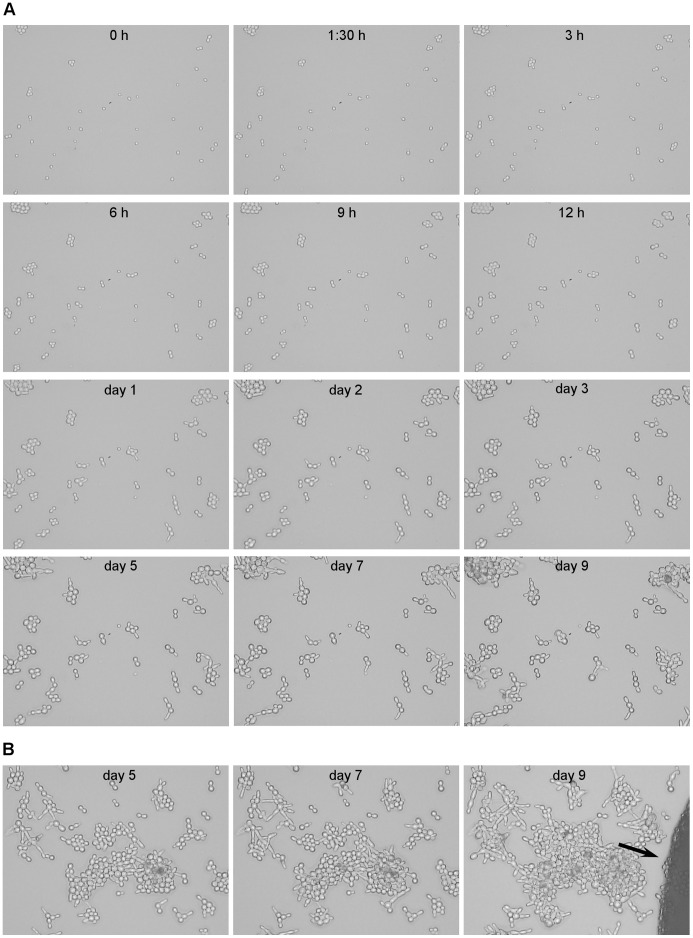
Follow up of *S. cerevisiae* microcolonies during prolonged exposure to 5-FC. Follow up of *S. cerevisiae* microcolonies during prolonged exposure to 5-FC for the specified time. After inoculating a 90 mm diameter Petri dish containing SC medium with 100 µg/ml 5-FC in the same fashion as performed for SLAM experiments, a 6 cm^2^ slice was cut out and placed on a glass slide and kept in a moist chamber. Random fields were chosen at time 0 and followed through time as specified in the Methods section for the times indicated in each picture. Panel A shows detailed follow up of one field. Adjacent to the field shown in panel B, a cell acquired resistance and grew, eventually invading the followed up field (black arrow). A marked difference can be observed between the edge of microcolonies and the 5-FC^r^ colony.

Only one gross chromosomal rearrangement was found among 50 5-FC^r^ clones examined by CHEF (Fig. 3). Instead, the origin of resistance was mostly found by sequencing the above mentioned genes. When examining the mutations in what we have counted as primarily resistant clones (pre-exposure resistance), we observed a marked difference between those that grew within 2 days and those that took 3 days to grow (Table 2). Indeed, all 6 resistant clones that appeared within 2 days showed a point mutation in the deaminase FCY1, 4 nonsense and 2 missense. Of note, 3 of the nonsense mutations were the same transversion 58 CG→AT. In contrast to what we expected, the spectrum of mutations of the clones that appeared at day 3 after drug exposure was drastically different from that at day 2. Indeed, all 5 mutations found were in the permease FCY2 gene. Moreover, most (4 of 5) were nonsense transversions, 3 TA→AT and 1 TA→GC, and we found no transitions.

**Figure 7:**
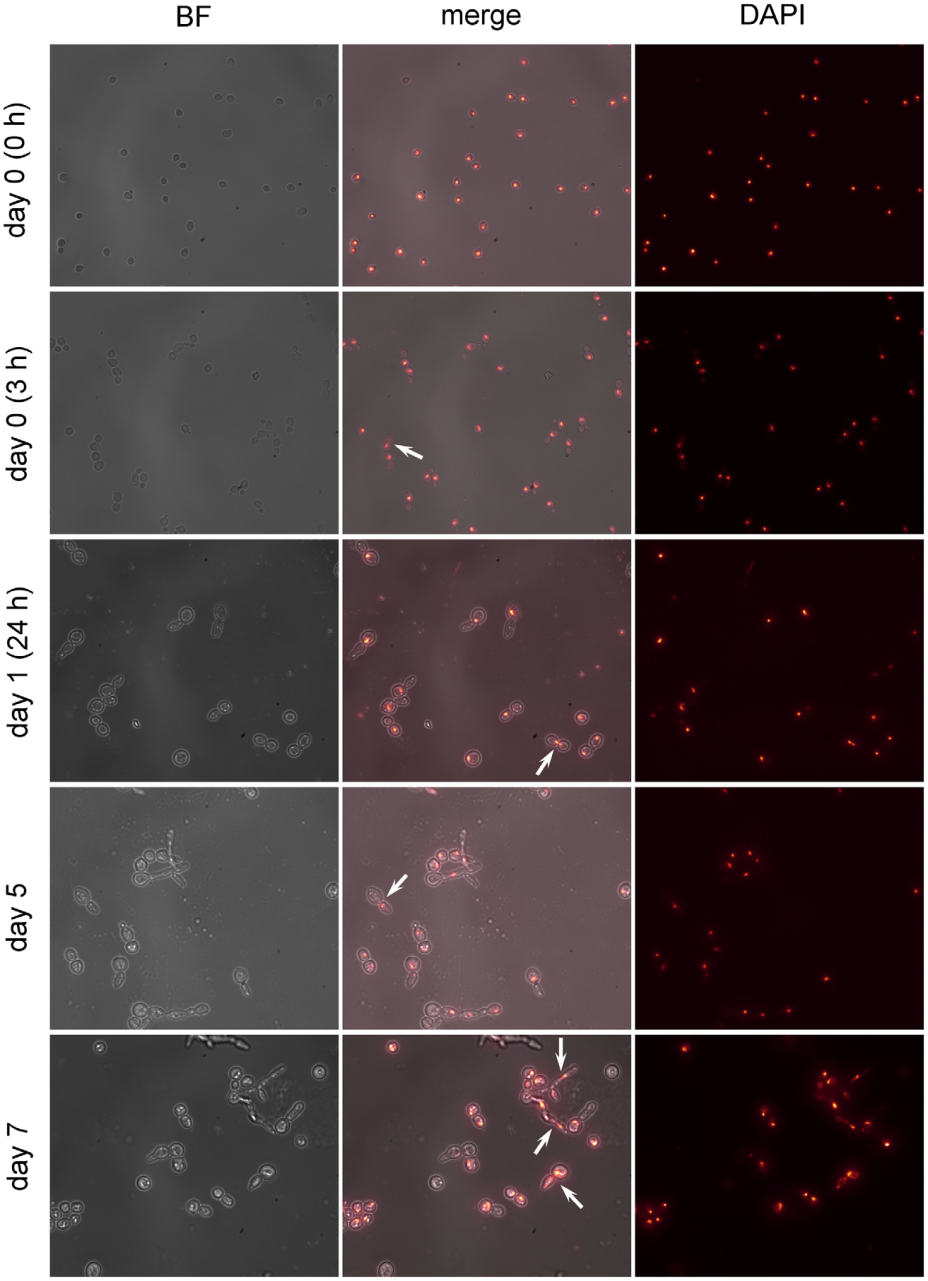
Fluorescence microscopy of cells exposed to 5-FC for long term. Effect of 5-FC on nuclear segregation of *S. cerevisiae* after exposure for the specified time. For each panel, resistant colonies were excised from one 90 mm diameter plate and the remaining non-resistant cells were washed off with sterile H_2_O and kept at −24°C. On the day of the experiment they were dyed with DAPI and examined by fluorescence microscopy. Left column was obtained using bright field; right column was obtained using fluorescent illumination; central column was digitally obtained by merging the other two. Arrows point to observed anaphase events.

When searching for the origin of resistance in those clones emerged after day 3, we could not find the causative mutation in 5 (9.1%) of 55 5-FC^r^ clones (Table 2). Of these 5 clones, 1 presented a silent substitution in FCY1, while the remaining 4 presented no mutations in any of the 4 genes sequenced. Overall, in those 55 clones sequenced, we found a total of 59 mutations (2 concomitant mutations in 9 clones analysed). The vast majority of the mutations were found in the FCY2 gene. Indeed, of 59 mutations, 54 (91%) were in the FCY2 gene, belonging to 49 clones (89.1% of the analyzed clones). Aside from FCY2, we found 4 point mutations in the deaminase FCY1 gene, belonging to those clones that displayed 2 concomitant mutations. We also found 1 transition in FUR1 - concomitant with a mutation in FCY2 -, and no mutation at all (silent or otherwise) in CDC21. The spectrum of the 59 mutations was distributed as follows: 2 (3.4%) insertions, 7 (11.9%) deletions, 11 (18.6%) transitions and an equal number of transversions. The remaining 28 (47.5%) mutations were produced by intrachromosomal (since our strain is haploid) homologous recombination with FCY22 (Fig. 4), a 1592 bp gene located 8459 bases downstream of FCY2, in chromosome V, with 87% homology to FCY2. The mechanisms for this recombination warrant further research.

**Figure 8:**
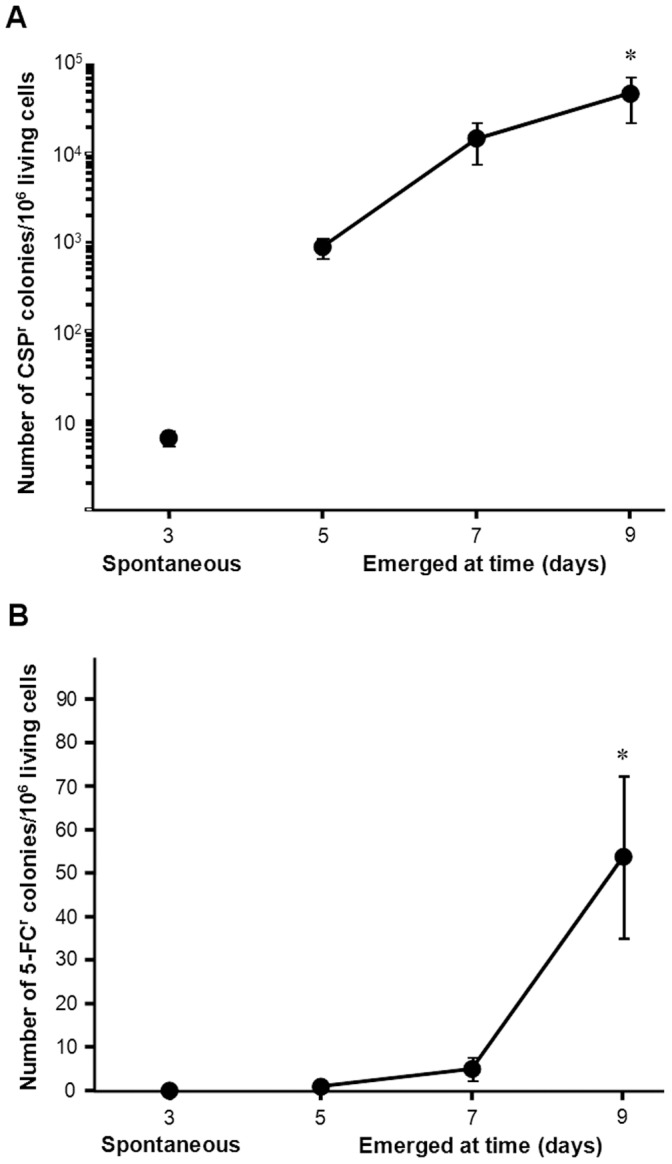
Kinetics of the acquisition of resistance by yeasts during prolonged incubation on drug-containing agar media. **Panel A**, 5·10^6^ *S. cerevisiae* cells (N = 11) were inoculated on SC agar medium supplemented with 0.72 µg/ml CSP. **Panel B**, 5·10^5^ *C. albicans* cells (N = 21) were inoculated on agar medium containing 100 µg/ml 5-FC. SLAM experiments were performed as described in Materials and Methods. Mean and standard error for N independent subpopulations (as specified). * indicates p<0.05 as evaluated by ANOVA with Bonferroni post-hoc test.

### Analysis of the Cell Cycle

In view of the differences found in the sequencing data, we pondered over the possibility that the non-resistant living cells remaining on the plate are replicating their DNA and that the mutations are arising from replication or repair errors. As figure 5 shows, the vast majority of the non-resistant living cells remaining on the SC+5-FC plate seem to undergo an arrest in G1/S phase already after 3 hours exposure to the drug, lasting for at least, 6 days. Nevertheless, a small subpopulation of cells undergoing a regular cell cycle (as occurs in tumors) or cells undergoing a very slow cell cycle would be undetectable by FACS. Thus, we performed a time-lapse microscopic examination (Fig. 6).

**Figure 9:**
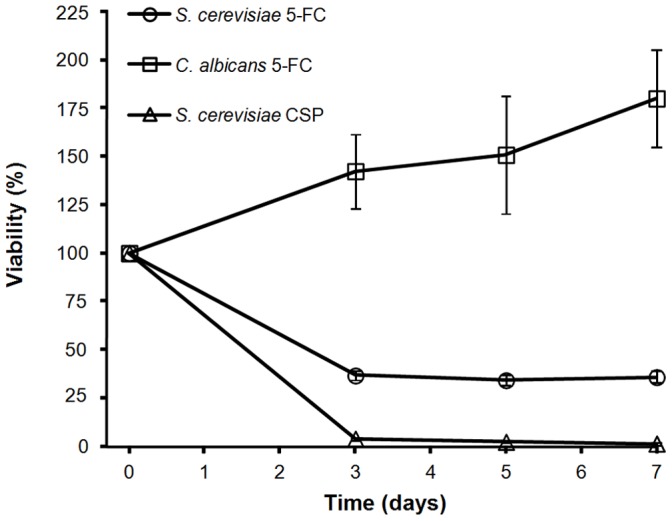
Effects of antifungal drugs on yeast viability. Five hundred thousand *S. cerevisiae* (circles, N = 39) or *C. albicans* (squares, N = 21) cells were inoculated on agar medium containing 100 µg/ml 5-FC. Five million *S. cerevisiae* (triangle (N = 11)) cells were inoculated on SC agar medium supplemented with 0.72 µg/ml CSP. Viable colony forming units were determined as specified in figure 1B. Survival is shown relative to the number of viable cells present 3 hours after plating on drug (as explained in the Methods section) and is the mean and standard error for N independent subpopulations.

As figure 6 shows, cells grown in drug-free liquid medium finish the already initiated cell cycles after they are plated in presence of drug, so they nearly double the population within the first 3 hours. After that, the cell division is quite slow, taking from several hours to days for a cell to bud. During 7 days we followed up the budding events of 182 individual cells present in the culture after 3 hours (to avoid including previous cycles started in absence of drug), failing to observe any individual cell performing several cell cycles. Instead, we observed that 56% of those 182 cells present on the slide after 3 hours carried out further budding events at later times, definitely a larger number than just a small subpopulation of cells. When observed through time, it is apparent that the cycle is extremely slow and that a small bud can take from several hours up to days to grow. We can thus conclude that no small population is undergoing a regular cell cycle. Rather, this data together with that collected from FACS analysis suggests that the cell cycle is slowed down to a great extent at an early S phase, with or without transient arrest.

While performing this time-lapse microscopy we noticed a large number of aberrant shapes and apparent pseudohyphae. We wondered whether those aberrations possessed a nucleus or if they arose during failed attempts to divide. We used fluorescence microscopy to examine the cell nucleus (Fig. 7), observing that the aberrant cells do have a nucleus, so there is cell division, albeit abnormal. We also observed that mitosis is indeed progressing, since we found instances of anaphase (arrows).

### Extension of the Model of Acquisition of Resistance through Adaptive Mutations

In order to evaluate whether our model is specific of drug or of organism we assayed a more relevant clinical drug, CSP, on the one hand, and an infectious yeast, *Candida albicans,* on the other hand.

When examining the acquisition of resistance (Fig. 8), we observed that upon prolonged exposure to CSP, many more resistant *S. cerevisiae* colonies emerged than upon exposure to 5-FC (Fig. 8a). Indeed, while in the 11 subpopulations analysed the spontaneous mutation frequency (6.4·10^−6^±1.2·10^−6^ colonies/viable cell) was similar to that of 5-FC, the acquired resistance frequency rose to 4.7·10^−2^±2.7·10^−2^ colonies/viable cell (p<0.05).

The spontaneous mutation frequency of *C. albicans* leading to 5-FC^r^ was within the expected range (8.4·10^−8^±8.4·10^−8^ colonies/viable cell) [14] (Fig. 8b), but by day 9 the resistance frequency rose to 5.4·10^−5^±1.9·10^−5^ colonies/viable cell, more than 600 fold the spontaneous frequency (p<0.001). A total of 21 subpopulations were evaluated in these experiments.

In both instances we performed reconstruction experiments to examine the stability of acquired resistant phenotypes, finding similar results to those with *S. cerevisiae* and 5-FC.

Analyzing these data, we queried what kind of effect, if any, these drugs were exerting on yeast viability (Fig. 9). In spite of the high mutation frequency found in *S. cerevisiae* when in presence of CSP, the drug had a severe fungicidal effect, so that we had great methodological difficulties in counting the non-resistant living cells remaining on the plate on day 7, a number that was beyond our limit of detection on day 9. In contrast, as described in the literature, the effect of 5-FC on *C. albicans* was fungistatic rather than fungicidal, cells nearly doubling (180.0±25.3%) their original population after 7 days.

## Discussion

Acquisition of resistance secondary to drug treatment is a major clinical problem in both bacterial and fungal infections as well as in cancer [4], [10], [15]. One way cells can acquire resistance is by changes in a gene of the route of action of the drug, i.e. mutation. Adaptive mutations are those that enable a cell to adapt to a growth-limiting (stressful) environment [6]. Several adaptive mutation mechanisms are used by bacteria to acquire resistance to various antibiotics [1], [2], [4], including the SOS or the RpoS-controlled stress responses.

Data about adaptive mutation mechanisms in eukaryotes is rather scarce. A few reports suggest their existence in certain types of cancer (see, for instance, the works by Hara et al. [16] and Steinkamp et al. [17]) and most research about yeasts has been performed on *S. cerevisiae* (reviewed in [6]). The models available are based on nutrient deprivation, where one of these models has revealed the requirement of yKU70, a non-homologous end-joining protein [18]. Nevertheless, in addition to lacking clinical relevance, these models are rather limited, since they are based on an artificial yeast construction that can only adapt to starvation conditions by introducing a frameshift in a promoter region, preventing the researcher from detecting any other types of mutations that may be acquired by the cell. All these considerations suggest that adaptive mutation processes do take place in eukaryotic cells [19], but a broader model amenable to genetic studies is needed to describe them in depth. This need has prompted us to develop the presented model.

A model to study adaptive mutation has to meet two main criteria, as reviewed by Heidenreich [6]. First, “a population of cells has to be kept in a prolonged state of growth limitation by the application of nonlethal stress conditions”, ideally arresting completely the cell cycle. “Second, the possibility should exist that, as a matter of principle, the proliferation arrest is abolished by mutations… The resulting emergence of a mutant clone amidst the majority of” non-growing “cells allows the detection of adaptive mutation events”. The model we have developed meets both criteria:

1. *S. cerevisiae* cells have slowed down their cell cycle to such an extent that one single cell may take from several hours to days to complete one cycle and bud, as shown above. Thus, one would think that most of the mutations arisen seem to emerge not only due to errors by replicative DNA polymerases, but from other mutagenic mechanisms as well.
2. The proliferation arrest is abolished by a number of possible mutations, as already detailed in the introduction. Thus, as the second requirement states, mutations do exist that abolish the proliferation arrest.

When comparing our model to the available starvation models, we find some similarities. For instance, the late arising resistant colonies result from post-plating mutations. This can be concluded from the results of the reconstruction experiments and from the observation that each mutation found in the vast majority of the colonies analyzed was absent from both the spontaneous mutant colonies from the same subpopulation and from the other late arising colonies of the same plate, which clearly shows that the mutation occurred after plating. Analysis of the mutation spectrum shows a clear distinction between the early and the late arising mutations, which proves that whatever mechanism is taking place, it is clearly different between the two phases. Whereas the early arising (spontaneous) clones show mostly point mutations in either FCY1 or FCY2, the late arising clones (adapted) predominantly have recombined FCY2 with the 8 kbase downstream 87% homologous gene FCY22. In regard to the point mutations, the types and genes found varied within the different phases. Whereas the vast majority of point mutations found in spontaneous clones were in FCY1 (day2) or transversions in FCY2, in adapted clones we found predominantly recombinations and a similar number of transitions than transversions in FCY2. Of 50 clones analyzed, we only found by CHEF 1 gross rearrangement, although smaller rearrangements would remain undetected by this method.

Comparing our mutation spectrum to that found in the already existing models we have found no similarities. We already expected that because of the different nature of the mutations necessary to escape the proliferation arrest. Whereas in starvation models only a frameshift in a specific gene (Lys2 or Hom3) would get selected and re-start growth, our model is very little stringent with the type of mutation that may become selected and fixed. Frameshifts, fragment or gene deletions, point mutations, recombinations, could all provide the mutation necessary to overcome the growth limitation, as we have seen.

Of note, we found not even one mutation in CDC21, even though it has been proposed that a change-of-function point mutation in this gene might confer resistance to 5-FC [8]. This underscores the metabolic need for this gene and the difficulties in finding a point mutation that would enable the enzyme to work without being affected by the drug.

Our model presents additional advantages over the starvation-based models. Firstly, it is a closer approach to the clinical situation and has more clinical relevance:

1. In recent years *S. cerevisiae* has acquired relevance as model organism in areas such as pharmacology and oncology. Although it lacks some aspects inherent to cancer, the biochemical routes for the organism functioning are highly conserved in eukaryotes, from yeast to human. Most genes present a high sequence homology, and protein functions are enormously conserved among species [20]. Therefore, using *S. cerevisiae* as model allows us to discover the role of proteins within the cell metabolism context and to identify therapeutic targets.
2. The cytosine analogue 5-FC, failed after its development as an antitumor drug, proved useful as an antifungal drug. However, it is no longer used as a single agent due to the high frequency of secondary resistance acquisition (30%) [8]. Still, 5-FC is currently being explored in new approaches to cancer therapy (see for instance [21], [22]).

In addition to its clinical relevance, another advantage of our model is that the adaptive mutation frequency has similar characteristics to those obtained in other models [23], [24], but is orders of magnitude higher, which facilitates the assays. Nevertheless, to rule out the existence of an intrinsic mutagenic effect of 5-FC, and to evaluate its broadness and clinical relevance, we have validated it with two variants.

The first variant is using the infectious yeast, *C. albicans*, as model organism. This clinically important fungus can cause life-threatening systemic infections, especially in immunocompromised patients [25]. When compared to the haploid *S. cerevisiae* strain used, the spontaneous mutation frequency in this diploid organism was two orders of magnitude lower (in the range of 10^−8^ mutants/viable cell). But in agreement with the clinical situation, the adaptive mutation frequency increased greatly through time, becoming similar to that of *S. cerevisiae* (in the range of 10^−5^ mutants/viable cell). In contrast to *Saccharomyces*, viability data show that the *Candida* population is nearly doubled within 7 days. This difference is probably due to the different effect of 5-FC on either species (fungicidal for *S. cerevisiae* vs. fungistatic for *C. albicans*). Some cells of both species are slowly reproducing but, while some other *S. cerevisiae* cells die, the *C. albicans* ones don’t die, remaining quiescent (static), so the overall number of viable cells increase. The slow replication during our assays imply that the adaptive mutation frequencies can be, at least in part, introduced by replicative polymerase errors, but since the extent of this replication is so small, some other mechanism(s) may possibly be underlying too.

The second variant of our model was using an echinocandin drug, CSP. This antifungal agent was approved for use barely 12 years ago, with a cost of over 500 USD per day per patient. Its target is completely different from that of 5-FC, since it is a noncompetitive inhibitor of β(1–3)-glucan synthase, an enzyme that catalyzes the extracellular synthesis of β(1–3)-glucan of the cell wall [10]. Although CSP elicited a spontaneous mutation frequency similar to 5-FC, the adaptive frequency was extremely higher, reaching a range of 10^−2^ CSP^r^/viable cell. There are some reports on the emergence of CSP resistant candidiasis upon preemptive therapy, as well as of isolates with reduced susceptibility to CSP during therapy, but as of yet, data on the frequency or the incidence of this secondary resistance is not yet available, although it will be very interesting to compare our in vitro data to it.

In this model, we have observed that yeasts acquire heritable resistance upon prolonged exposure to drugs with either fungistatic or fungicidal effect, and unexpectedly, the more fungicidal the effect, the more the adaptive mutation frequency is increased over the spontaneous. This suggests that a factor(s) may exist that leads to adaptive mutation and that this factor increases during cellular stress, and the stronger the stress, the higher the factor increases. Whether this factor is a metabolite (e.g. increased oxidants that may be damaging the DNA) or a biochemical mechanism (analogue to bacterial adaptive mechanisms like SOS response, or an increased DNA damage by an endonuclease, for instance) is currently under study. What we have observed is that spontaneous mutations in our *S. cerevisiae* –5-FC model are different from adaptive mutations, which implies different mutagenic mechanisms. We have shown here that one of the adaptive mechanisms is through recombination events of the permease FCY2 with an 87% homologous gene, FCY22. These observations pose a number of interesting queries, like what are the factor(s) leading to the observed increased mutagenesis (metabolites and/or proteins), or why we have not found recombinations with FCY21, another highly (77%) homologous gene 2 kb closer to FCY2 (6 kb downstream), or finally, whether these types of recombinations are of use during evolution.

The following model would fit our data anpd that available from other authors:

Upon exposure to the drug, as to other stress types, cells become transiently arrested in G1 [26]. Very slowly, they would struggle to progress through G1 and start S phase. During this struggle, either in G1 or in early S (or in both) they would be accumulating DNA damage and breaks (SSB and/or DSB). Then, some of the DSBs would get repaired while others may persist through S-phase and the duplicated sister chromatids. Since both would be harboring a DSB at the same position, they could be jerry-rigged repaired in G2 [27]. Some of these repairs may lead to the ectopic intrachromosomal recombination observed (probably by the Rad52 epistasis group), some may be performed by the other DNA repair systems (MMR, etc), some may perhaps lead to cell death. Once all the breaks are repaired the cell finishes its cycle. If no incorrect repair leads to a resistance-conferring mutation the daughter cells struggle again through slow G1, and the process starts again. If one repair leads to a mutation that confers resistance, the daughter cells are no longer sensitive to the drug and thus can grow normally. Eventually all the cellular resources remaining are spent in this burdensome cell-cycling and the cell enters a chaotic situation of “duplicate and mutate, or die” that we can see by FACS analysis at day 10 of exposure or by morphologic analysis at day 9.

In summary, we have presented a model for adaptive mutagenesis with the following characteristics:

1. It fulfils the requirements of slowing cell cycle and detectable mutation events described by Heidenreich [6].
2. It is amenable to genetic studies, and will enable the characterization of proteins involved using knock-out yeast strains, transcription studies, etc.
3. It is a clinically relevant model, with applications both to cancer and to yeast invasive infections, and may be useful for studying acquisition of resistance to drugs as long as such resistance can be achieved by mutation.
4. An ectopic recombination with a homologous gene takes place with a high frequency.

Further studies on the FCY2:FCY22 recombination mechanism, the spectrum of mutations that lead to CSP resistance, what proteins are involved in this adaptive mutation, and the possible accumulation of mutagenic metabolites are necessary in order to describe adaptive mutation in eukaryotes.
